# Lectures on the Diagnosis and Treatment of Diseases of the Lungs

**Published:** 1841-04-17

**Authors:** W. W. Gerhard


### MEDICAL EXAMINER.

DEVOTED TO MEDICINE, SURGERY, AND THE COLLATERAL SCIENCES.

No. 16.] PHILADELPHIA, SATURDAY, APRIL 17, 1841. [Vol. IV.

### LECTURES ON THE DIAGNOSIS AND TREATMENT OF DISEASES OF THE LUNGS.

BY W. W. GERHARD, M. D.

### LECTURE XIII.—(*Continued.*)

#### PHTHISIS—TREATMENT.

The treatment of phthisis is by many regarded as never curative, but merely as a means of palliating the most severe or harrassing symptoms of the disorder. If we apply the term consumption only to those cases in which the disease is far advanced, and the constitutional deterioration is extreme, it is very plain that no means of cure exist, and that even palliation is in many cases difficult; but if we speak of consumption as of other diseases which tend to a fatal termination only after having passed through their early and more curable stages, it is strictly curable, and, like these disorders, must be treated in different ways, according to the mode of its development; for as tubercles are attended with very different symptoms, and originate in various modes, it is very clear that the most opposite methods of treatment may prove efficacious in combatting the affection in its forming stage. But after the tuberculous deposit has fairly commenced, it obeys its own laws of growth, and presents the secondary symptoms, such as hectic fever, emaciation, &c., which are peculiar to itself, and then one uniform method of treatment is desirable, or at least seems indicated. Besides, although the modes of development of tuberculous disease are very numerous, there is a form in which the symptoms are regular and uniform; and even in those varieties in which the modes of origin are most unlike, there is a peculiar character impressed upon the various symptoms, which is dependent upon the scrofulous or tuberculous diathesis.

This treatment would be specific for the disease, and would be curative if it could cause with certainty the absorption of the secreted product, and favour the cicatrization of cavities, when the loss of substance was not extremely great. If we possessed such a mode of treatment, we might then, with great confidence, expect to cure phthisis in nearly every stage. But as no such specific exists, we are obliged to content ourselves with the administration of alteratives, which have but a limited influence on the growth of tubercles, and of such remedies as act either upon the causes of the disease, or on the accidental disorders which favour the tuberculous deposit in an indirect way.

The alteratives used in phthisis are, for the most part, such as exercise a tonic and invigorating influence, at the same time that they produce their proper effect as alteratives. Mercury is always injurious as a direct remedy in phthisis; it can never be of service except in those cases in which there is decided inflammation, and the tubercles result directly from it; but even in this class of cases, the influence of the remedy is certainly injurious so far as it affects the proper tuberculous disorder, and it must be discontinued as soon as the inflammatory symptoms are removed. The effect of mercury in phthisis is now so well known that it has almost become an axiom in medicine to avoid it in the treatment of this disease. Iodine is much more used than any other alterative; and if employed with discretion, it scarcely ever does harm. I have found it beneficial at the commencement of cases in which the fever was but moderate, and the local inflammation but slight, especially when a circumscribed chronic bronchitis has preceded for a long time the actual development of tubercles. Hence it is well suited to those cases which are preceded by chronic inflammation of the trachea and larger tubes, and pass slowly into phthisis, and to the cases which are most closely connected with external scrofula. The patient is then often robust in appearance, and the local disease is slow in forming. Iodine is also useful in the purely constitutional cases, provided it be given cautiously, and the emaciation of the patient has not advanced very far; it should then be combined with vegetable tonics. The preparation to which, from habit, I have restricted myself, is Lugol's solution, prepared of the strength directed in the United States Pharmacopœia,—that is, one scruple of iodine, and two of hydriodate of potassa, to seven drachms of water. Of this I give to an adult from three to six drops two or three times daily; I scarcely ever exceed six drops three times daily, and often give much less. For the good effects of the medicine may be obtained much more certainly in this way than by giving it in larger, but more irritating doses. I have never witnessed any other mischievous effects from the iodine than the disorder of the digestive canal, and a fulness of the head which sometimes results from it, but it is very certain that in some rare cases it acts as other powerful alteratives occasionally do, and it may enfeeble or disturb the functions of the whole body without removing the morbid action. Hence it is advisable to discontinue its use from time to time, and resort to mild purgatives for a few days, or to abstain totally from all medicine until the tone of the stomach is restored; it then is scarcely possible that an injurious result should follow. As the action of iodine is slow, we cannot observe any immediate impression produced by it; but when it is acting well, the complexion and strength of the patient improve, and the cough at the same time gradually diminishes. The latter effects may be promoted by appropriate expectorants, which should be given at the same time with the iodine. The appetite and strength almost always increase; and if these fail, or become less, instead of increasing, it is almost a sure indication that the medicine is not acting well. It is often useful to administer laxatives from time to time, even if the medicine be not suspended. As iodine evidently acts merely as an alterative, it is beneficial in that condition of the economy which precedes the secretion of tubercles, as well as in their more advanced stages, and it may be conjoined with other alteratives, such as the compound decoction of sarsaparilla, or with mild tonics. Without attributing to iodine any specific virtue, I am quite convinced that its powers are very great in commencing phthisis, and that it often effectually arrests the progress of the disorder. The remedy seems to me to be least adapted to those cases in which tuberculization is very rapid, or the inflammation of the serous membranes very acute.

There is no other alterative of a medicinal kind to which I attach much importance. There is none to which I could refer as possessing enough certainty of action to make it useful in the majority of cases of phthisis: the mineral alteratives are more or less irritating and depressing in their effects; and the vegetable, although they are, in some cases, of service, cannot be relied upon with much certainty. They are evidently most beneficial where there is a constitutional deterioration which is going on very slowly, and rather precedes than actually accompanies the deposit of tubercles,—that is, it is the same disease which has not yet reached its highest point; and although the knowledge we possess of the virtues of this class of medicines is as yet extremely limited, there is great reason to believe that they may possess considerable power in arresting the early stages of constitutional phthisis. Most of the alteratives now used for this purpose are at the same time tonic, such as the preparations of sarsaparilla and the compounds of rhubarb with the bitter tonics, or with soda. In my own practice I resort to these remedies, chiefly to replace the iodine, or to aid its action.

When phthisis has fairly commenced, iodine, or any other alterative, is designed to favour the absorption of a product which is actually deposited. But there are many cases in which we know that a tuberculous action is going on; that is, that the process which ends in tuberculous secretion is actually at work. It is then important to arrest it in its forming stage, and iodine is often of benefit in these cases. It is true that direct proof of this is extremely difficult, because it is not easy to prove that a disease which is slow and obscure in its mode of formation, is really influenced or not by any remedy. The reasoning must be probable, and not demonstrative; and the truth is approached more or less nearly as the observer possesses the proper abilities for drawing conclusions of this kind. In these forming cases of tuberculous disease, iodine seems to act like alteratives of a hygienic character, and is certainly useful if no directly injurious consequences result from it; but it must be given in small doses, and from time to time should be intermitted. If these cases be very acute, the remedy must be omitted; for as a general rule it is quite unsuited to either the forming or the formed cases of acute phthisis or any other inflammatory form of tuberculous disorder. In these acute cases the inflammatory element predominates, and the action of the remedy is too stimulating, as it is in cases of phthisis which begin by local inflammation. With these reservations as to its use, iodine is the most efficient remedy in early and in forming phthisis. How far its usefulness extends, is not a subject upon which we can speak with entire confidence.

In advanced cases of phthisis hectic supervenes; and iodine and all other alteratives are useless, unless they act merely as tonics. Indeed, iodine has generally appeared to me to be of positive injury as soon as softening had taken place. For even if its influence upon those portions of the lungs in which the disease has not advanced very far is good, it acts injuriously upon the surface of cavities and the softened tubercles.

In the early stages of tuberculous disease of the lungs, hygienic alteratives have always claimed the first place; indeed, you may readily believe that no medicinal alterative can well be useful if the hygienic measures which are best adapted for the disease be neglected. These are very well understood; and, besides the choice of proper localities for a residence, and for a journey or sea-voyage, consist mainly in adopting such precautions, and in pursuing such a course of life as is least fitted to develope the disease.

This part of our subject leads us naturally to the examination of some of the causes of phthisis. When we remember the circumstances under which the disorder occurs, we may divide them them into two classes—those of a general and those of a local character. The general causes are such as exist originally in the individual, or arise from the circumstances in which he is placed; the latter are those which may be to a great extent obviated by art, and the action of the former may thus be checked indirectly, or at least not favoured. The local causes of phthisis are either directly inflammatory, or at least belong to morbid conditions which must be removed by medicinal rather than by hygienic measures. If the general causes include an hereditary predisposition to tuberculous disease, it is of course necessary to insist the more strongly upon those that are accidental. You find these causes enumerated in the work of Dr. Clark, and in most others upon the subject, and it is not necessary to enter much into detail upon this subject; some classification, however, may be adopted, to render the same intelligible.

1. We may place hereditary predisposition in the first instance. This is universally admitted, and the strength of it is increased if the parents were actually labouring under the formed disease at, or a short time previously to the birth of the child. It may descend from either parent; but it would seem that the mother exercises the greatest influence in this respect, especially if she nurses the child herself. In other respects the usual laws of hereditary transmission hold good, and the probabilities of their action are increased if the child present the character of the scrofulous temperament.

2. Depressing causes which debilitate the powers of life, increase the tendency to the morbid action. These, of course, are very numerous. Imperfect diet, exclusion from light and fresh air, and mental depression, are amongst the most powerful. Inaction, or a diminished activity of body, favours the same result. These causes are very obvious in patients admitted into hospitals with other chronic diseases, and afterwards attacked by phthisis. The reverse of these causes is always to be advised, and whenever practicable the greatest attention should be paid to them. One of the advantages of a journey certainly arises from its invigorating influence, and the abundant supply of healthful air which is thus obtained for the patient. The depressing causes often arise from the effects of disease which is cured, but leave the patient in an enfeebled state: this is often the case with typhoid fever; in other instances it produces a more direct impression, and the phthisis supervenes before the fever entirely ceases.

3. Certain occupations are known by direct observation to favour the development of consumption; these are such as require a constrained position, and especially sedentary confinement in close rooms. Mineral or vegetable dust or powders diffused in the atmosphere contribute to the same result. Hence the propriety of changing a pursuit is often a matter of strict necessity. Irregular exposure to cold and heat has a similar tendency, but it is much more effective as a cause of the accidental inflammations that often precede phthisis.

Although these are the chief of the general causes of phthisis, the list might be much extended; they are, however, more or less analagous in their character, and are more or less directly depressing upon the individual.

The alterative effects of a long journey and of change of residence, are well known in phthisis. They both act nearly in the same way: a journey in a pleasant season of the year, or in a climate which renders all seasons agreeable, is often of great benefit in forming phthisis, or in those varieties of the disease in which there is not much febrile excitement or local inflammation; if these exist, the journey is irritating, instead of invigorating. If the strength of the patient is good, the journey should be made on horseback, or in an open carriage, and be pursued as long as the strength of the patient continues to improve. A sea-voyage is sometimes preferred to a land journey; as a general rule, however, it is less useful; but there are cases in which the strength of the patient is not great, but the disease at the same time is slightly advanced, and the fever moderate, in which a sea-voyage in a mild latitude is of great benefit. It is also of great benefit in those cases in which the phthisis is attended with slight, but frequent hæmoptysis during its early stages. A short voyage is of little comparative benefit; it should be long enough to act as a decided alterative; hence, one to the East Indies, or to the Mediterranean, or South America, answers best. The shorter voyages to Madeira, or the West Indies, are only advisable as necessary to a winter's residence in these climates.

The question of a change of residence is always of great interest to a phthisical patient; in fact, there is no one upon which he is more disposed to consult his medical adviser. The general anxiety felt by patients to resort to this mode of relief, is a conclusive proof that there is something in it, for it still continues when the lapse of years shows that the advantages of such a residence are excessively overrated. These advantages may be stated very briefly; by a winter's residence in a warm, but equable climate, the tendency to slight congestions or inflammations of various portions of the organs of respiration is obviated, and a cause of irritation is then removed. Secondly, the mildness of the climate allows the invalid to enjoy the advantages of fresh air and exercise without much discomfort or risk. Lastly, the change of climate and of air is of itself of great benefit as an alterative. These advantages are, however, limited; they are not specific in the treatment of consumption; hence many cases are not at all relieved, some are even aggravated. If the disease be of the acute form, and especially if it be attended with much fever, the patient is almost always rendered more feverish by the journey, and the affection tends to advance more rapidly; or if the disorder be so much advanced that the strength of the patient is rapidly declining, no advantage can be expected. It is in the milder and more chronic cases that the change of air does good, especially if the patient has found by experience that the winter is of injury to the organs of respiration, and give rise to much cough or other signs of laryngeal or tracheal irritation. Of this class of patients very few individuals will be found to die abroad; most of them return with some benefit, especially for the first winter; if the disease be not arrested, however, the benefit of a second winter is very doubtful. When the disorder of the digestive organs is very prominent, the benefit from the voyage is very considerable, but that from a protracted residence in a warm climate is very doubtful.

The advantages resulting from a change of climate are not, therefore, such as to induce us to advise patients to leave their homes, and subject themselves to many privations without due consideration; and we should steadily oppose it, if the reasons for the voyage are not strong.

It is difficult to point out the precise spot which is most suitable for the winter residence of a consumptive patient. Many physicians differ with perfect good faith as to the relative advantages of the different places which they recommend. The island of Cuba, Santa Cruz, the West India islands in general, and Florida, are most in fashion with invalids from the United States. Madeira is much resorted to by those from England, and, to some extent, by Americans; and various parts of the South of France, of Italy, and the shores of the Mediterranean generally, are preferred by the continental nations. A full account of the various advantages of many different situations will be found in the work of Sir James Clark, to which I may refer you. My own advice is regulated very much by the peculiar circumstances of the patient, his willingness or his desire to undertake a distant sea-voyage, his pecuniary means, &c. All of the different localities have some advantages; perhaps, at present, the island of Cuba offers more than any other. But it is not expedient to recommend any one situation to the exclusion of others,—still less is it expedient to advise a change of residence, even for a season, or a change of occupation, except upon strong grounds, and in cases where no harm at least will ensue.

